# Renal Artery Blood Flow and Surface Parenchymal Perfusion During Renal Artery Endoshunting in a Porcine Model

**DOI:** 10.1016/j.ejvsvf.2024.10.003

**Published:** 2024-10-11

**Authors:** Johan Millinger, Marcus Langenskiöld, Andreas Nygren, Klas Österberg, Joakim Nordanstig

**Affiliations:** a Department of Molecular and Clinical Medicine, Institute of Medicine, University of Gothenburg, Gothenburg, Sweden; b Department of Vascular Surgery, Sahlgrenska University Hospital, Gothenburg, Sweden; c Institute of Clinical Sciences, Sahlgrenska Academy, University of Gothenburg, Gothenburg, Sweden; d Department of Anaesthetics and Intensive Care Medicine, Sahlgrenska University Hospital, Gothenburg, Sweden

**Keywords:** Aortic aneurysm, Endovascular procedures, Laser Doppler flowmetry, Perfusion, Vascular surgical procedures

## Abstract

**Objective:**

Ischaemia and reperfusion can result in permanent tissue damage. During complex open abdominal aortic surgery, transient clamping of the renovisceral arteries may be required to successfully complete the vascular repair. Endovascular shunting (endoshunting) presents an alternative technique for managing such temporary renovisceral ischaemia. This study aimed to investigate the performance of endoshunting to the renal circulation in a porcine model.

**Methods:**

This study of five domestic pigs investigated arterial volume flow rates during endoshunting of a single renal artery and the associated impact on renal perfusion parameters (laser Doppler renal parenchymal perfusion, renal oxygen extraction, and selective urinary output). The study was performed in three steps: baseline registrations (30 minutes), endoshunting (120 minutes), and restoration (60 minutes). The right kidney was used as the experimental side and the left kidney as control.

**Results:**

The median arterial flow rate in the left control kidney remained constant throughout the experiment. On the right (endoshunted) side, the baseline median arterial flow rate was 267 (range, 160–404) mL/min. Following activation of the endoshunt, the median arterial volume flow dropped by 59%–110 (range, 45–150) mL/min (*p* = .018). During endoshunting, the median kidney surface perfusion decreased to 42% of the baseline value. On the control side, a rise in the median parenchymal perfusion was observed after endoshunt activation, which was again normalised following restoration of native right renal artery flow. During endoshunting, the median regional urine production was 0.32 (range, 0.12–0.50) mL/hour but resumed after renal artery flow restoration.

**Conclusion:**

On average, the endoshunted kidneys showed a rapid restoration of blood flow, parenchymal perfusion, and urine production after 120 minutes of endoshunting. This suggests that endoshunting to the kidney using an endoshunt system might be a promising strategy to preserve renal function when temporary interruption of native renal artery blood flow is needed during complex vascular surgical repairs involving the renal arteries.

## Highlights

• Endoshunting is a safe alternative for renal perfusion during vascular surgery.
• The kidney shows rapid restoration of urine production after 120 minutes of endoshunting.
• Endoshunts can be adapted for various vascular diameters.
• One benefit of endoshunting is the possibility of shunting from a percutaneous access.

## INTRODUCTION

While ischaemia and reperfusion can result in permanent tissue damage in most organs, reperfusion of ischaemic tissue can also lead to remote systemic injuries affecting multiple organ systems. In complex cases of suprarenal aortic pathologies requiring open surgical repair, there may be a need to temporarily occlude the visceral and or renal arteries to facilitate the vascular repair. For example, it is not uncommon to temporarily interrupt arterial flow to one or both renal arteries during open repair of juxta- and pararenal abdominal aneurysms.^1^

Endovascular shunting presents an alternative technique for managing this issue in various vascular surgical scenarios. This technique enables shunting of blood from percutaneous or minimally invasive arterial access (e.g., with cut down) while maintaining some degree of arterial flow in the donor artery during the shunting period. This department has used the brachial artery as a suitable donor for shunting both renal and visceral arteries during complex open abdominal aortic repairs. In a recently published study, it examined the experimental flow capacity of several endovascular shunt systems and compared them with the flow rates observed for the widely used 9 F Pruitt-Inahara carotid shunt, for which flow capacity is well defined.^2^^,^^3^ The study demonstrated that endovascular shunting techniques could achieve comparable volume flow rates to the 9 F Pruitt-Inahara carotid shunt. An *in vivo* study was thereafter carried out in a porcine model, where endoshunting from the infrarenal aorta to the lower extremity was tested under *in vivo* circumstances. In this animal model, the endoshunt system appeared to provide adequate perfusion to prevent significant striated muscle ischaemia.^4^

This study, using a pig model, aimed to investigate how the volume flow rates in the main renal artery affect key renal function parameters (laser Doppler kidney surface parenchymal perfusion, selective kidney oxygen extraction, and selective urinary output) during endovascular shunting into a single renal artery.

## MATERIALS AND METHODS

### Overall study design and animal model management

This exploratory study was conducted on five domestic pigs that were approximately three months old with a weight of 44–60 kg. The study was approved by the local ethics committee for animal studies at the administrative court of appeals in Gothenburg, Sweden (Ref. nr: 5.2.18-7209/17). The study was conducted in accordance with the guidelines of the European Parliament directive and the report written following the ARRIVE guidelines.^5^ The investigations were performed at the University of Gothenburg's Experimental Biomedicine facility, which is an approved animal research facility. The pigs were premedicated with Zoletil/Dexdomitor in the stables and thereafter transported to the operating theatre, and subsequently placed under general anaesthesia. Anaesthesia was induced by administration of Metacam 0.4 mg/kg intravenously and Vetergesic 0.03 mg/kg intramuscularly. Anaesthesia was maintained with a continuous administration of isoflurane (Attane vet, VM Pharma AB, Stockholm, Sweden). Infusion of crystalloids and intermittent administration of small doses of phenylephrine (0.1 mg/mL) were used when needed to sustain a stable blood pressure, with a mean arterial pressure target of 80 mmHg throughout the procedure.

### Detailed description of the experiment

The right kidney was used as the experimental side, while the left kidney was used as an internal control. The left kidney was perfused via the native left renal artery without interruptions throughout the experiment. Through a midline laparotomy, the abdominal aorta and both renal arteries and veins were dissected, and the anterior surfaces of both kidneys were exposed (to allow subsequent renal surface laser doppler probing). An intravenous bolus of heparin (200 IE/kg) was given at the initiation of the experiment and thereafter was administrated as an hourly dose of 100 IU/kg for the duration of the experiment (i.e., higher than regular human dosing to account for the hypercoagulability of the blood of domestic pigs compared with humans^6^). The investigation was performed in three steps:

1. **Baseline measurements:** All direct and indirect measures of left and right renal perfusion (see points one to six below) were performed during 30 minutes of baseline sampling.
2. **Endoshunting period**: Following the initial measure-ments, the donor introducer to the endoshunt system, an 8 Fr Prelude Short Sheath introducer (Merit Medical, UT, USA) was placed in the infrarenal aorta using the standard Seldinger technique. Fig. 1A shows the setup of the interconnected shunt system. The 8 F Pruitt-Inahara shunt was used as the distal component as it was the most appropriate off the shelf component in this hospital that could fit the smaller diameter of the porcine renal artery. The right renal artery was then clamped proximally and a transverse renal arteriotomy was performed in the proximal part of the right renal artery. The endoshunt was then introduced into the renal artery and activated while confirming its proper function (Fig. 1B). During the 120 minutes of right renal artery endoshunting that followed, all indirect and direct measurements of renal perfusion were recorded continuously for both the right (endoshunted) and left (internal control) kidneys. Selective oxygen extraction was only measured across the right kidney circulation.

**Figure 1:**
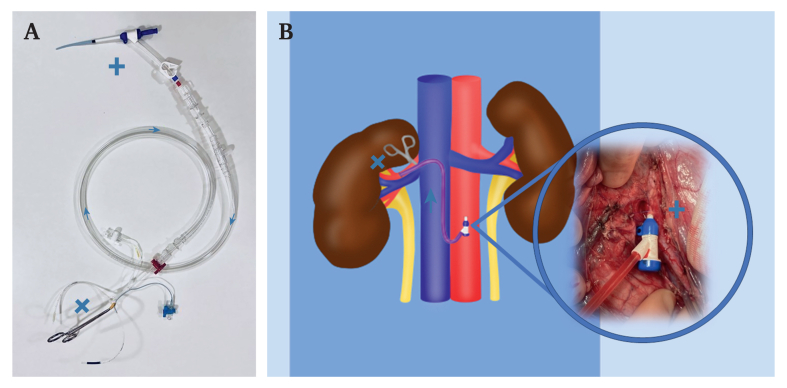
Endoshunt system. A) Image showing the system constructed from an 8 F Prelude short sheath introducer (Merit Medical) (+), 30 cm long quarter inch perfusion tubing (Sorin Group) (arrows indicate direction of flow), and an 8 F Pruitt F3 carotid shunt with T-port (LeMaitre Vascular, Burlington, MA, USA) (X). B) Schematic overview of the endoshunting stage of the experiment: (+) the endoshunt system inserted in the infrarenal aorta, arrow indicates the direction of flow in the endoshunt system (purple), (X) vascular cross clamp of the right renal artery, the distal component of the endoshunt inserted in the right renal artery distal to the vascular clamp.
3. **Flow restoration**: The shunt was removed after 120 minutes of right renal artery shunting, and the right renal arteriotomy was closed with interrupted vascular sutures and the native right renal artery blood flow was reactivated using standard surgical techniques. Thereafter, all measures of renal perfusion were measured with the same time intervals during a restoration phase that lasted 60 minutes. Following completion of the restoration phase, the animal was euthanised with an injection of 80 mg Allfatal vet. (100 mg/mL).

### Measurements of renal blood flow and perfusion

The following parameters were measured to assess renal artery and renal tissue blood flow during the experiments:

1. Renal artery volume blood flow: a MiraQ Vascular system (Medistim, Oslo, Norway) with conventional QuickFit arterial probes was used to determine renal artery blood flow in both renal arteries. Renal artery flow rates were recorded every five minutes throughout the experiment.
2. Endovascular shunt tube volume blood flow: an HT110 Flowmeter (Transonic Systems Inc., Ithaca, NY, USA) capable of measuring plastic tube blood flow was used to measure blood flow delivery to the endoshunted (right) renal artery every five minutes.
3. Kidney surface parenchymal perfusion was measured continuously with a laser Doppler flow probe (0.25 mm fibre separation, 780 nm wavelength (Perimed AB, Järfälla, Sweden) that was sutured to the anterior kidney surfaces on both sides. The average tissue perfusion during each 30 minutes of the different phases of the experiment was calculated both in dedicated perfusion units and as observed percentage changes from baseline.^7^
4. Kidney oxygen extraction in the endoshunted (right) kidney was measured by arterial and venous blood gas sampling every 30 minutes. The arterial blood gas was collected from an arterial line in the carotid artery, whereas the renal venous blood gas was measured in a selective catheter placed directly into the right renal vein (i.e., enabling calculation of oxygen extraction rates from the right kidney).
5. Urinary output (mL/min) from the left and right kidneys was measured throughout the experiment in four of the five animals, using catheters selectively placed in the left and right ureters.
6. The mean arterial pressure was measured continuously during the entire experiment via an arterial line placed in one of the carotid arteries and the average mean arterial pressure every five minutes was filed.

### Statistical methods

Descriptive summary statistics are presented as median (range) or relative frequencies as appropriate. The Mann–Whitney *U* test was used to compare independent variables of interest, whereas the Wilcoxon signed rank test was used for pairwise comparisons. A *p* value of <.050 was considered statistically significant. Calculations were performed in IBM SPSS Statistics 29 (Armonk, NY, USA).

## RESULTS

### Left and right renal arterial blood flow rates

**Figure 2:**
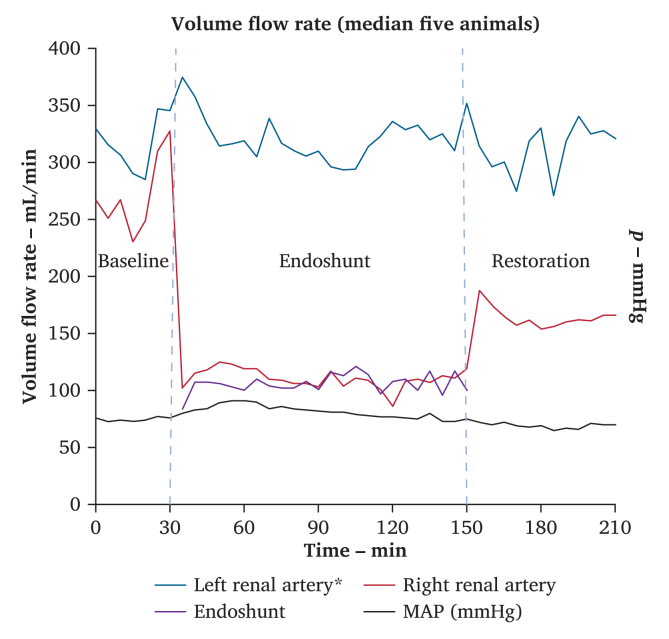
Arterial volume flow rate and blood pressure during the three phases of the experiment (based on median values from five animals); ∗measures based on recordings from four animals.

The median arterial volume flow rate in the left control kidney remained relatively constant during all three parts of the experiment (baseline phase, 316 (240–503) mL/min; endoshunting phase, 318 (202–485) mL/min; and restoration phase, 319 (165–517) mL/min). On the experimental right side, the median arterial volume flow rate was slightly lower during the baseline phase at 267 (160–404) mL/min. The median arterial volume flow rate dropped 59% to 110 (45–150) mL/min (*p* = .018) following activation of the endoshunt. The recorded arterial volume flow rate in the endoshunted right renal artery was comparable when directly measured on the native renal artery distal to the endoshunt and when directly measured over the plastic tubing of the endoshunt using the HT110 Bypass Flowmeter (median value during the endoshunting period was 107 *vs*. 110 mL/min; *p* = .189). After removal of the endoshunt during the restoration phase, the median arterial volume flow rate in the right renal artery increased to 162 (74–292) mL/min but remained 39% lower than baseline throughout the restoration phase that lasted 60 minutes (Fig. 2).

### Renal parenchymal perfusion

**Figure 3:**
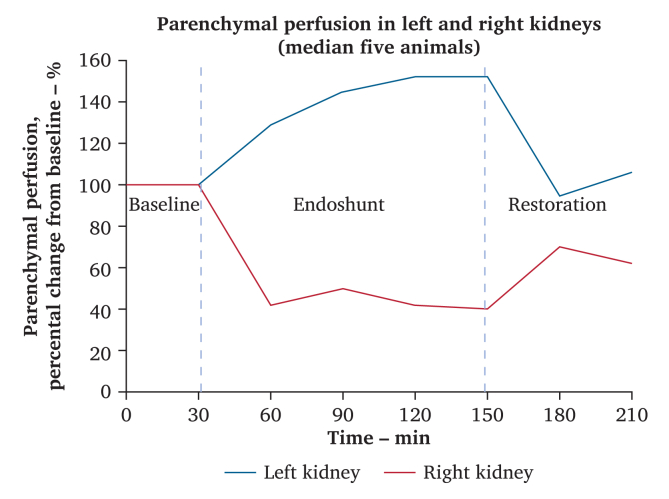
Kidney parenchymal perfusion, percental change from baseline. Left kidney *n* = 4, right kidney *n* = 5.

At baseline, the average kidney surface perfusion was comparable between the experimental right kidney and the internal control left kidney (178 *vs*. 181 perfusion units). Following the insertion of the endoshunt on the right side, the median right kidney surface parenchymal perfusion rate initially decreased to 42% of the baseline value. Over the course of the 120 minute endoshunting period, the median parenchymal perfusion rate remained constant. On the left internal control side, a consistent rise in the median parenchymal perfusion rate was observed immediately after endoshunt activation. The median parenchymal perfusion rate in the left control kidney had reached 152% of the baseline level by the end of the endoshunting phase. During the restoration phase, the median parenchymal perfusion rate on the experimental right side rose to 70% of the baseline level, while the corresponding perfusion rates returned to baseline levels on the left (Fig. 3).

### Oxygen extraction

**Figure 4:**
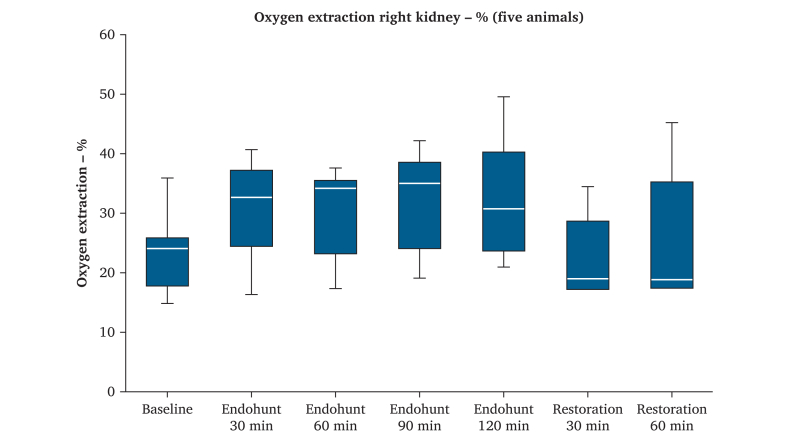
Oxygen extraction rate of the right kidney, at baseline and at 30 minute measurement intervals during the endoshunting - and restitution periods. Boxes represent upper and lower quartiles, bar represents median, and whiskers represent range.

The mean oxygen extraction rate over the experimental right kidney was 24% at baseline. There was an immediate increase in the median oxygen extraction rate to 33% following activation of the endoshunt. The oxygen extraction rate was constant during the endoshunting period. The median oxygen extraction rate of the right kidney returned to 19% after 60 minutes of native artery flow restoration, a level similar to the baseline observation (Fig. 4).

### Urine output

**Figure 5:**
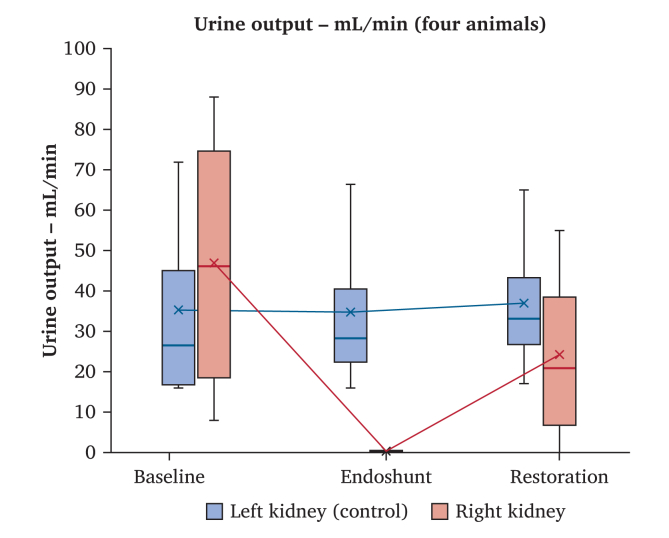
Selective urine output from the right (experimental) and left (control) kidneys, X represents mean urine output, boxes represent upper and lower quartiles, bar represents median, and whiskers represent range.

The median urine production from the left control kidney remained relatively constant throughout the experiment (Fig. 5). The median urine production was also comparable between the left and right kidneys during the baseline measurements. However, the median urine production from the right kidney decreased to low levels (0.32 [0.12–0.50] mL/h) following endoshunt activation. Urine production resumed in the experimental right kidney in three of four research animals during the restoration phase (Fig. 5). In the animal where urine output did not resume, there were technical difficulties to place the distal part of the endoshunt, which is why the elapsed time from right renal artery clamping to proper endoshunt activation took 43 minutes.

### Exam protocol deviations

There were technical difficulties in introducing the distal part of the endoshunt into the renal artery in one animal, leading to a delay of 43 minutes from renal artery clamping to endoshunt activation. The placement of selective catheters in the left and right ureters was omitted in another animal, which is why selective urine output could not be measured in this animal.

## DISCUSSION

One kidney was perfused through an endoshunt for 120 minutes in this experimental animal study. Despite observing some degree of hypoperfusion in the endoshunted kidney, evidenced by reduced blood flow and minimal urine production during the endoshunting phase, the overall renal perfusion delivered by the endoshunt appeared to have exceeded a critical threshold that avoided permanent ischaemic damage to the kidney. This was supported by the subsequent restoration of native renal artery blood flow, parenchymal perfusion, and urine production when concluding the endoshunting period.

Baseline kidney perfusion showed an average volume blood flow rate of about 300 mL/min; this is within the blood flow range that has previously been reported for human renal arteries.^8^^,^^9^ During this test of an endoshunt system, the blood flow through the renal artery perfused by the endoshunt decreased to about one third of the observed baseline arterial blood flow, which led to an accompanying decrease in the kidney surface parenchymal perfusion of approximately 50%. This was most certainly related to the inherent flow restriction of the 8 F carotid shunt used as the distal component in the interconnected endoshunt system. The anatomy did not allow a larger endoshunt distal component in the porcine model that was used. However, from experience, the adult human renal artery normally accommodates a 9 Fr Pruitt-Inahara carotid shunt, which would reduce this diameter restriction of the distal endoshunt component.^8^

The regulatory mechanisms of renal blood flow are complex and not fully understood but include the complicated interplay between both intrinsic renal factors as well as systemic endocrine and paracrine factors involving the renin–angiotensin system, vasopressin, prostaglandins, adenosine, and nitric oxide release, but also renal sympathetic nerve activity.^10^, ^11^, ^12^ In this experiment, the control left kidney demonstrated no increase in renal artery blood flow or urine production during the endoshunting period, but cortical perfusion increased, suggesting a redistribution of tissue perfusion within the kidney. This may partly be explained by a systemic response triggered by the decreased flow in the endoshunted kidney.^11^ During the 120 minutes of endoshunting, the oxygen extraction rate in the endoshunted kidney increased from 24% to 33%, and the endoshunted kidney ceased producing urine, two obvious signs of relative hypoperfusion. However, on restoration of native blood flow to the endoshunted kidney, the transient increase in parenchymal blood flow observed in the contralateral kidney rapidly reverted to close to baseline levels. Additionally, three of four research animals resumed urine production from the endoshunted kidney within one hour of re-established native blood flow.

These findings imply that the effects of hypoperfusion were considerably less pronounced than what would have been anticipated after 120 minutes of severe ischaemia and thus suggest that the endoshunt protected from severe kidney ischaemia.^10^^,^^13^^,^^14^ In the fourth research animal there were technical difficulties in placing the distal part of the endoshunt and, as a result, the kidney had global ischaemia for 43 minutes. This animal did not resume urine production, suggesting that the elapsed ischaemia time before effective endoshunt activation might have been too long. This is supported by previous observational studies in open aortic surgery, where ischaemia times exceeding 30 minutes were associated with decreased renal function post-operatively.^14^

When the endoshunt was removed and native blood flow was subsequently restored, the median arterial blood flow in the endoshunted renal artery also did not completely return to baseline levels within the restoration observation window of 60 minutes (Fig. 2). This phenomenon of decreased renal perfusion during renal reperfusion might be caused by spasm in the renal artery, but also by increased renal vascular resistance. The exact mechanisms behind the increased vascular resistance within the kidney are unknown.^12^

From a clinical standpoint, the benefits of endoshunting include the possibility of shunting from a percutaneous or otherwise minimally invasive access without complete disruption to the blood flow in the donor artery. Furthermore, endoshunting techniques can bridge both short and long distances between any donor and recipient artery and, depending on the precise components used, it can also easily be adapted to various vascular diameters.

In comparison with other strategies used when performing open surgery on complicated aortic aneurysms requiring aortic clamping above the renovisceral arteries, endoshunting has obvious benefits to the clamp and go strategy by extending the presumably safe time frame for the surgical repair well beyond the 30 minutes. The safe duration of renal artery endoshunting remains unclear but given the initial observations in this study, 120 minutes appears to be a relatively safe threshold that should be more than sufficient to conclude the most complex open abdominal aortic procedures.

### Limitations

This study had some limitations. One obvious limitation was the species differences between porcine and human renal anatomy and renal artery diameters. There were technical problems in inserting the endoshunt in the renal artery in one of the research animals due to the small diameter of the vessel. This phenomenon occasionally also manifests in human patients, serving as a caveat associated with the method. Nevertheless, from a technical standpoint, all other techniques that rely on some sort of shunting also hinge on the capacity to introduce a shunt in the renal artery. The sample size of five research animals made it difficult to draw robust statistically significant conclusions from the observed changes. Additionally, the regional oxygen extraction was not measured on the control kidney side, which in retrospect would have been interesting and could have led to a more comprehensive understanding of the overall kidney haemodynamics during endoshunting of a solitary renal artery. Furthermore, renal blood flow and cortical perfusion were measured but perfusion of the renal medulla was unable to be measured due to the tissue penetration depth of the laser Doppler technique; therefore, the relative perfusion distribution within the kidney was not studied. No completion angiography was performed after removal of the endoshunt and the subsequent suture of the renal artery. Finally, neither systemic nor regional ischaemic markers (such as lactic acid) nor kidney function laboratory parameters were measured during the experiments.

### Conclusion

The main finding in this study was that the endoshunted kidney demonstrated a rapid restoration of native renal artery blood flow, parenchymal cortical perfusion, and urine production following 120 minutes of endoshunting. Consequently, this suggests that endoshunting to the kidney using an endoshunt system might be a promising strategy with which to preserve renal function when temporary interruption of native renal artery blood flow is required during more complex vascular surgical repairs involving the renal arteries.

## Conflict of interest

None.

## Funding

This study was supported by grants from the Swedish state under the agreement between the Swedish government and the county councils, the ALF agreement (ALFGBG-785741 and ALFGBG-822921) and the Swedish Heart–Lung Foundation (20190194 and 20200258).
